# Collectanea Medica: Consisting of Anecdotes, Facts, Extracts, Illustrations, &c.

**Published:** 1825-11

**Authors:** 


### COLLECTANEA MEDICA:

CONSISTING OF

ANECDOTES, FACTS, EXTRACTS, ILLUSTRATIONS, &c.

*Relating to the History or the Art of Medicine, and the Collateral Sciences.*

Floriferus, ut apes, in saltibus omnia libant,

Omnia nos, itidem, depascimur aurea dicta.

### ART. I.—*Extracts from "Lectures and Observations in Medicine."* By the late MATTHEW BAILLIE, M.D.

WE should not have thought ourselves at liberty to insert any part of this volume without having obtained permission: this, however, has kindly been granted us. The *unpublished* writings of the late eminent Dr. BAILLIE must naturally excite great interest; and we are happy that it is thus in our power to gratify our readers.—EDITORS.

THE following papers are printed for private distribution among the friends of the late Dr. Baillie, by his executors, in obedience to directions left in the following codicil to his will; dated Cavendish-square, Dec. 27, 1821.

"I wish my two Introductory Lectures to the course of Anatomy which I gave in Great Windmill-street, my Lectures upon the Nervous System, read before the College of Physicians, and a short account of my experience in the Practice of Medicine, to be *printed,* but not *published.* One hundred and fifty copies may be printed, of which one copy may be given to each of my more intimate medical friends, and the remainder to the Royal College of Physicians in London. They are hardly of sufficient importance to be published, and yet I am unwilling that they should be completely lost, as something useful may be extracted from them. They will form together a small octavo volume."

### *Some brief Observations drawn from my own Experience upon a considerable number of Diseases.*

I have now practised as a physician for more than thirty years, and have, for the greater part of that time, been so much occupied with visiting patients, that I have seldom been able to write notes of individual cases. It has occurred to me, however, that some advantage might be derived from my leaving a short record of the results of my experience in a considerable number of diseases.

I am convinced that the most successful treatment of patients will depend upon the exertion of sagacity or good common sense, guided by a competent professional knowledge; and not by following strictly the rules of practice laid down in books, even by men of the greatest talent and experience. It is very seldom that diseases are found pure and unmixed, as they are commonly described by authors; and there is almost an endless variety of constitutions. The treatment must be adapted to this mixture and variety, in order to be as successful as circumstances will permit; and this allows of a very wide field for the exercise of good common sense on the part of the physician. A physician who should be guided strictly by the rules laid down in books, would be a very bad practitioner. In the following short observations on the treatment of various diseases, I shall state impartially the result of my experience, without entering into any speculative reasoning, which is often very fallacious.

*Hanger Hill; July 22,* 1819.

### *Complaints of the Head.*

Many persons of both sexes are affected daily with headaches, of more or less severity, for many months, and often for some years. They chiefly prevail towards the middle time of life, but occur often at an earlier period. They may take place in any part of the head, but are more commonly felt in the forehead, or over one eye, or in the back part of the head. Such headaches I have found in general to be very little benefited by bleeding, either general or topical. In the accounts which patients have given me of the effect of this remedy, they have said that they have either received from it no benefit at all, or that it has lasted but a few hours; or that the headaches have even been worse after cupping, or the application of leeches. I have generally found such headaches to be most benefited by temperate living, great attention to avoid improper diet, purgative medicines, and bitters. The best common medicine is rhubarb and soap, in such doses as to give two motions daily. A few grains of calomel, with an aperient draught, such as an infusion of senna with a drachm or two of Epsom salts given occasionally,—as, for instance, once in a fortnight or three weeks,—are sometimes of much use. A due degree of exercise taken daily, both on foot and on horseback, is likewise in some cases very serviceable. Some headaches I have known relieved by nervous medicines, but not frequently. In some cases this complaint is relieved by no plan of medicine or management whatever, but will gradually, after some months or years, subside. The seat of such headaches is, I believe, in the scalp, and not in the inside of the cranium. They depend chiefly for their cause upon the state of the stomach and bowels, or upon an irritable state of some of the nerves of the scalp. In most headaches of severity, it is right to make one or two trials of the effect of topical bleeding; but not to persevere in the repetition of this measure for many months, as is often done, even though it produce no benefit.

The cutting the hair of the scalp very short, and the application of cold, by a large sponge wrung out of cold water and applied to the upper part of the head, will often give great temporary relief when the skin has been previously hot.

*Apoplexy.*—This disease, in its most severe form, depends commonly upon blood being poured out into the substance of the brain from some ruptured blood-vessel. This generally takes place in the medullary substance, near one of the lateral ventricles, but it may occur in any part of the brain. The milder forms of apoplexy depend upon a distention of some of the vessels of the brain, from an undue accumulation of blood in them. I have known, however, one instance of fatal apoplexy where many of the blood-vessels were found, upon examination after death, to be much distended with blood, but no blood had been extravasated in any part of the brain.

The chief remedy in apoplexy is large bleeding, to be repeated according to circumstances. Topical bleeding by cupping and leeches is likewise often of use. The next remedies in importance are purgative medicines of considerable power, and acrid glysters. The head should be kept high or elevated, and cold may be applied with advantage to the top of the head. If the patient should recover by these means, the best plan of management, in order to escape from another attack, is to live almost entirely throughout future life upon vegetable food, and to abstain from wine, spirits, and malt liquor. It will be of considerable advantage to avoid any strong or long-continued exertion of the mind. In a few instances, when the full state of the vessels of the brain had for some time subsided, I have derived considerable advantage from the moderate use of tonic medicines, and more especially of steel.

*Hydrocephalus.*—I have known in my own experience but one instance of this disease being cured, when fully formed. In this case all the symptoms were well marked, and the disease had made such progress that squinting and an irregular pulse had taken place. There had been no peculiar treatment, except that mercurial ointment was applied daily to a considerable sore on the upper part of the head, which had been produced there by a blister. The individual is now alive, and is a young lady of good talents, which she has highly cultivated.

I have seen a few cases, in which there appeared to be a strong threatening of hydrocephalus, that got well by the application of leeches and blisters to the head, and brisk mercurial purges; but I cannot determine whether these cases, if less actively treated, would have terminated in true hydrocephalus or not.

*Epilepsy.*—This disease appears to me to have become much more frequent within the last twenty years than formerly. If this remark be generally true, it may perhaps be accounted for by the progress of luxury, which must render the nervous system more irritable. I have known very few instances of epilepsy radically cured; but a considerable number of cases in which the intervals between the attacks have been rendered much longer. The medicines which have appeared to me to have most influence in removing or retarding the attacks of epilepsy have been the argentum nitratum, viscus quercinus, and the oleum succini. Of these, the first is the most powerful; but, when it has been used for a good many months, it tinges the skin of some individuals of a dark colour. I have known two instances of this effect from it in my own experience. The bowels, too, should always be kept open, and the effect of brisk purgatives should be tried in the beginning of the disease.

It is of great use, in the treatment of epilepsy, that the patient should live very temperately, and should avoid every thing which may tend suddenly to excite or to harass the mind. Patients should eat animal food sparingly, and should abstain from wine, ale, and porter altogether. The hair should be cut short, and cold applications should be applied to the head whenever the skin of it feels hot. This management is often of much use in rendering the attacks both less frequent and less violent. The causes producing epilepsy are various; but I believe that in this disease there is constantly a tendency to a greater accumulation of blood than is natural in the vessels of the brain.

*The Tic Douloureux.*—The tic douloureux seems to me likewise to have become more common of late years, and I think it is more frequent among men than women. I do not recollect to have seen any instance in which it has been permanently cured, either by internal medicines or by an operation. I have known some instances of its being cured for a time (that is, for several months, or even a year,) by medicines; and those which have appeared to me of most use are Peruvian bark and arsenic. The operation of dividing the nerve has in some instances prevented a return of the disease for one or two years, but has not, as far as I know, prevented it permanently. The courage and patience under suffering in this complaint, displayed by some individuals, have been truly astonishing.

### *Of some Diseases of the Neck.*

The most common disease in the neck is the swelling of one or more lymphatic glands. This is most apt to take place in young persons who have fair complexions and delicate constitutions. It is always a very tedious disease, and is seldom much benefited by medicine. The remedies which I have found of most use have been sarsaparilla combined with soda, Peruvian bark combined with soda, and some form of steel. These medicines will, however, often have but a very imperfect influence upon the complaint. Sea air and tepid sea-water bathing are often beneficial; but I think that the air and waters of Malvern are more useful than any other remedy. I have known a good many cases which had been but little improved by the common remedies, and by a residence upon the sea-coast, with all its advantages, which have afterwards got quite well by the patients residing three or four months at Malvern.

*Bronchocele.*—This disease is not very uncommon in this country: it is more frequent among women than men, and much more so among young than old persons. It is not often much benefited by medicine, but will frequently disappear of itself. Sometimes the swelling grows, even in this country, to an enormous size; and I have known one or two cases in which the patient was destroyed by the swelling compressing the trachea and the œsophagus. The medicines which I have found of most use have been burnt sponge, soda, and mercury, used externally, either as an ointment or in the form of plaster.

*Chronic Inflammation of the Larynx and Trachea.*—This disease occurs frequently in this country, and, upon the whole, I think is more common among men than women. It is often confined to the inner membrane of the larynx and the upper part of the trachea; but frequently it spreads downwards, even to the inner membrane of the bronchia. This disease always continues several months, and often, with short intervals of amendment, for years. Not unfrequently it lays the foundation of future phthisis. Remedies generally produce only a very gradual influence upon the disease, and sometimes none at all. Benefit is not unfrequently derived in some degree from the repeated application, at short intervals, of leeches to the fore part of the neck, or the skin covering the upper bone of the sternum. The frequent application of small blisters to the same parts will occasionally be of use; but perhaps the most useful remedy is a small seton inserted under the skin of the side of the neck, very near the larynx. Internal medicines often produce very little good effect; but the medicine which I have found, upon the whole, to be the most beneficial, has been the extractum conii. I have sometimes directed five grains of it to be taken three times a-day for many weeks together, with manifest advantage.

*Of the Quinsy.*—I have but one observation to make with regard to this disease, which is of some little importance. It is usual to endeavour, throughout the course of it, to prevent suppuration from taking place, by the repeated application of leeches under the angles of the lower jaw. It is certainly very desirable that suppuration should be prevented, and that inflammation of the tonsils should gradually subside by resolution. I have found, however, by experience, that suppuration is by such means very often not prevented, but only that inflammation proceeds more slowly to this issue. Hence the patient suffers for a considerably longer time; and the suffering in this disease is often very great. If, therefore, one or two applications of leeches do not lessen materially the inflammation of the tonsils and velum pendulum palati, I should recommend the progress of the inflammation to be encouraged by the inhaling of warm vapour into the mouth, and the application of poultices to the external fauces. In this way the disease will go through its progress more quickly, and the patient will suffer much less.

### *Of some Diseases of the Chest.*

I have very little to say either with respect to pleurisy or peripneumony. The earlier, after inflammation has taken place in the pleura or in the lungs, that blood is taken away from the arm, the sooner will the disease be subdued. Blood should in these diseases be taken away largely, and, if necessary, should be repeated again and again after short intervals. All other remedies are insignificant in comparison of the abstraction of blood from the system.

When this remedy has not been applied early enough, nor in sufficient quantity, and an abscess has been formed in the lungs, which has burst, patients have, in the greater number of instances that I have seen, recovered but very slowly. Under these circumstances, the medical attendant has little to do but avoid mischief. The constitution should be moderately supported, without being too much stimulated. Moderate doses of myrrh, decoction of bark, or infusion of some bitter, are sometimes of use. Light animal diet, and even a little wine, are sometimes useful in such cases; but great care should be taken that no new inflammation be excited.

In the course of my experience throughout many years, I have known a few instances of abscess being formed in the lungs, without any previous pain in the chest, or difficulty of breathing, or observable fever. Such patients, upon some exertion of the body, or even without any exertion, have suddenly coughed up a considerable quantity (perhaps half a pint or more) of pus; and this has been to the patient the first intimation of disease. In such cases the inflammation of the lungs must have proceeded so slowly as to have produced little or no pain in the chest, and not to have alarmed the constitution so as to excite fever.

*Of Phthisis Pulmonalis.*—In the course of my medical experience, I have known one or two cases of patients who recovered from phthisis which was apparently fully formed. It is probable, however, that with regard to these cases I may have been mistaken; and that, if I had inquired with sufficient accuracy into their history, I should have found that they were small abscesses of the lungs, of a common, and not of a strumous nature.

I have known a good many instances in which persons threatened with consumption have recovered by going into mild climates, or even into Devonshire or Cornwall; but I do not recollect a single instance in which they recovered when the disease had decidedly been formed. Change of air should be adopted very early, in order to give it the best chance of success. Such a variety of accounts have been given by patients, and even by medical gentlemen, of the comparative advantage of one place over another abroad, that I have found it impossible to decide which is to be preferred. I am disposed, however, to think that Madeira, the Hyeres, some parts of Portugal, Malaga, Nice, and Naples, are the best. It is very possible that different places may suit better with the constitutions of different individuals; and this conjecture, if well founded, may explain the cause of there being such a variety of opinions upon the subject. A patient should, if possible, spend two or three successive winters abroad, in order to give the best chance of the disposition to the disease being subdued.

When no active inflammation is going on in the chest in phthisis, I have sometimes found advantage from patients being allowed to take a little white fish or light animal food at dinner. In a very few instances I have found benefit derived from their taking one, or even two, glasses of wine, diluted with water, after dinner; but wine is generally improper.

I have known of no medicine which has been of permanent and substantial use in phthisis; but I have sometimes found a good deal of temporary advantage derived from myrrh, from ammonia, and from light bitters united to the acetic acid. The frequent repetition of blisters, or a seton inserted under the skin in some part of the chest, are occasionally of considerable use.

*Of Hydrothorax.*—When dropsy of the chest does not depend upon any diseased structure of the heart or lungs, I have found it much more readily affected by medicine than ascites or dropsy of the ovarium. Not unfrequently, under these circumstances, I have known water of the chest relieved, or for a time cured, by medicine.

The medicine which I have found most beneficial, has been mercury combined with squills and digitalis. Five grains of the pilula hydrargyri, combined with one grain of the dried powder of squills, and half a grain of the dried powder of digitalis, given twice or thrice a-day, have, in many cases under my care, either very much mitigated or for a time removed the disease. There has been some advantage from the mercury affecting slightly the salivary glands. Squills and digitalis are by themselves much less efficacious than when combined with mercury.

I do not recollect one instance of hydrothorax being permanently cured, although I remember a good many cases in which the symptoms were repeatedly removed by the same means in the same patient.

Where the difficulty of breathing has been very great, and the legs and thighs have been much swelled from anasarca, I have known much relief afforded by a scarificator and small cupping-glass being applied above the inner and outer ankle of each leg; and I do not remember any mortification attacking these small sores. The difficulty of breathing in such cases probably depended in part upon the water accumulated in the cellular membrane of some parts within the chest, and this was gradually emptied through the small openings made in the skin of the legs.

### *Of Palpitation of the Heart.*

Palpitation of the heart may take place at any period of life; but it is more common at an early period than any other,—as, for instance, from fifteen to twenty-five years of age. Perhaps, too, it may be more common in females than in males; but of this I am not very certain. At an early period of life, it does not in general depend upon any diseased structure of the heart, but either on a morbid irritability of the nerves of this organ, or upon some imperfect state of digestion. When it takes place from either of these causes, it always continues for a long time, (often, more or less, for two or three years,) but at length generally subsides. Rest of body and quietness of mind are two of the chief means which contribute to remove this disease. All quick motion of the body, and more especially walking up ascents, increases the complaint, and should as much as possible be avoided. Every thing which tends to excite or harass the mind has the same effect, and should be shunned whenever it is possible. To rest of body and mind should be joined very temperate diet; and, when this general plan of management has been continued for many months, or perhaps for a year or two, the disease usually subsides. Digitalis has sometimes been useful in mitigating this complaint, but frequently it produces no good effect.

Where the palpitation depends, either altogether or chiefly, upon the state of the stomach, it is gradually removed by temperance, by improving the condition of the stomach, and by keeping the bowels free from costiveness. I remember one case in which palpitation of the heart had taken place, and had continued for six months, in consequence of gout having attacked this organ. In this case the palpitation ceased suddenly and entirely when the gout attacked one of the feet in a full and decided form. This person is now alive, and has continued generally in good health, although it be nearly twenty years since the attack of palpitation.

In some young persons, palpitation depends upon an enlargement of the several cavities of the heart, produced not unfrequently by rheumatism attacking this organ. This cause of enlargement of the heart was overlooked by the physicians of this country, till it was discovered by the sagacity of my esteemed friend, the late Dr. David Pitcairn. The enlargement, in general, goes on increasing till life is destroyed; but I have known two cases where the enlargement stopped at a certain point, the increased action of the heart in a great measure subsided, and the patients acquired a tolerable share of health. They are both now alive, and they have the prospect of living, with care, to the ordinary term of life. Such a fortunate issue is very rare; but the disease may be generally retarded in its progress by much rest of body, quietness of mind, and a very temperate mode of living. Wine and every other fermented liquor should be avoided; and the patients, under such circumstances, should live almost entirely upon vegetable food.

At the middle and more advanced periods of life, palpitation of the heart often depends upon a diseased structure of some of its valves. This condition of the heart does not admit of any remedy, but must gradually become worse, until life be extinguished. But the symptoms may be mitigated, and the progress of the disease retarded, by little exertion of the body, by great temperance, and by a few ounces of blood being occasionally taken from the arm.

*Angina Pectoris.*—This distressing disease almost constantly depends upon an ossification of the coronary arteries of the heart, and admits of no effectual relief from medicine. I have met with two cases, however, in the course of my medical experience, in which symptoms exactly resembling those of angina pectoris depended upon an imperfect digestion; and the patients ultimately recovered entirely, by correcting the disordered condition of the stomach.

### *Of Diseases in the Cavity of the Abdomen.*

*Ascites.*—With respect to this disease I have very little to say. When it depends upon a morbid state of any of the abdominal viscera,—as, for instance, the liver or the spleen,—it is never permanently removed, and very seldom even relieved, till the morbid condition of these viscera is cured, if this event should fortunately take place. Even where the viscera in the abdomen are sound, or at least cannot be discovered by an accurate examination to be otherwise, ascites is rarely, according to my experience, cured by medicine. The ordinary diuretic medicines, as squills and digitalis, have commonly very little effect upon it. The medicines which I think, upon the whole, to have most influence upon this species of dropsy, are supertartrate of potash and small doses of elaterium. In two, or perhaps three cases, during my medical experience, ascites has gradually got well without medicine, after the common remedies had been sufficiently tried and had failed. I can entertain no doubt, from some late publications, that taking away blood from the arm will often be a valuable remedy in ascites, where there has been too much arterial action, or even some degree of inflammation, in the early part of the disease: but of this remedy I have not had sufficient personal experience to enable me to appreciate its value.

*Inflammation of the Peritoneum.*—Where this disease has not been connected with any peculiarity of season, or any epidemical complaint, I have found it to be cured by bleeding and purging, like other inflammations. Upon the whole, however, I think that it has been more relieved by repeated applications of leeches, than by general bleeding. I do not wish it to be understood that general bleeding is of no advantage in peritonitis, for sometimes it produces the greatest benefit. I think, however, that, in most cases, more benefit will be derived from the repeated application of leeches, according to circumstances, than from a repetition of the general bleeding. The purgative medicines which have appeared to me to be of most value, are calomel and the neutral salts.

*Of some Affections of the Stomach.*—There is no complaint more common in this country than an imperfect condition of the functions of the stomach. This generally shows itself by more or less of flatulence, by acidity, by a bitter taste occasionally felt in the mouth, and often by some degree of costiveness. This condition of the stomach generally arises from something wrong in the quantity or quality of the food,—from anxiety of mind,—and from a due degree of exercise not being regularly taken. It makes its progress very gradually, continues always for some months, and often even, more or less, for years.

The first object of attention should be to remove, as far as possible, the causes which produce it. Every kind of food should be avoided which the patients may have found, from their own experience, to have disagreed with their stomach. Most commonly, animal food that is very fat, or much salted, or fried, is difficult of digestion, and should either be eaten very sparingly, or should be altogether avoided. Young and white animal food is in general more difficult of digestion than what is brown and of middle age. The vegetables which are eaten should be very well boiled, and should be taken sparingly by such persons as are subject to flatulence or acidity. The waxy potatoe is almost constantly very difficult of digestion, and in general should be avoided altogether. There should never be so much food taken at a time as to give the feeling of fulness or distention in the stomach; and, except under very particular circumstances, there is no advantage in eating oftener than three or four times in twenty-four hours. The best common beverage in disordered conditions of the stomach is water, or toast and water; and three or four glasses of wine may be taken at or after dinner, according to the habits of the patient, or other circumstances. That wine is to be preferred which agrees best with the stomach, of which he is himself the most competent judge. Daily exercise is almost constantly necessary in order to preserve good digestion. Riding on horseback is upon the whole the best, for it gives a motion to the abdominal viscera which no other exercise is capable of; but walking is also very useful. A combination of the two is preferable to either; for riding on horseback chiefly exercises the abdominal viscera, and walking chiefly exercises the limbs and the thoracic viscera. Anxiety of mind should be avoided, whenever it can fairly be done; but it is often impossible to take advantage of this remedy.

With respect to medicines, there are none for this complaint which can be called specific. The most beneficial, however, which I have known are rhubarb, and some form of bitter medicine combined with alkalies. Eight grains of rhubarb formed into pills with soap, taken every night at bed-time, and some bitter, as infusion of cascarilla, calumba, quassia, or gentian, with some grains of soda or potassa dissolved in it, taken in the morning and before dinner, will often be very useful in this kind of disordered stomach. These remedies should be continued for five or six weeks at a time, should be omitted for two or three weeks, and occasionally resumed. If the alvine evacuations should be considerably lighter in their colour, or much darker than natural, mercury, given in moderate doses, and not for so long a time as to injure the constitution, will often be of great use. The large and indiscriminate employment of mercury in complaints of the stomach has, I think, been often very hurtful. Where acidity has been particularly prevalent in the stomach, I have sometimes found it more effectually corrected by the diluted mineral acids than by alkalies. Ten or twelve drops of the diluted sulphuric or diluted nitric acid, mixed with an infusion of some bitter, and taken twice a-day, will sometimes be very beneficial in this condition of the stomach.

There is an affection of the stomach in which the digestion is very imperfect, and in which considerable quantities of a transparent viscid mucus is formed. This often produces nausea, and is occasionally brought up by vomiting. According to my experience, this condition of the stomach has been frequently little benefited by medicine; but sometimes I have found the tinctura benzöes composita of considerable use. A drachm of it may be taken, mixed with water and some mucilage of gum acacia, three times a-day.

There is another affection of the stomach, less common than the former, but far more serious,—viz. where the stomach throws up in large quantity a fluid like cocoa. A quart of this fluid will often be thrown up at a time; and this will frequently be repeated for many days together. This condition of the stomach is sometimes connected with a diseased state of the liver, but sometimes it is independent of it, there being, at least apparently, no disease in this latter organ. In several instances it has proved fatal; but in others, and especially in two cases which I recollect, the complaint subsided for several months at a time, and the persons enjoyed in the intervals tolerable health. This state continued many years, and the patients are still alive. In one case I had an opportunity of examining the condition of the stomach after death. It was very capacious, and was half-filled with this brown fluid, but did not appear to be at all diseased in its structure. The neighbouring viscera, as the liver and spleen, were (as far as I recollect) perfectly sound. The fluid would appear to be formed by a diseased secretion of the inner membrane of the stomach, without any apparent morbid structure.

This disease, according to my experience, is but very little influenced by medicine or by diet. In two or three cases, some benefit seemed to be derived from astringent medicines combined with moderate doses of opium,—as, for instance, from tincture of kino, or tincture of catechu, with a few drops of laudanum, taken three or four times a-day. The bowels should be at the same time kept free from costiveness.

In some cases the stomach will lose almost entirely the power of digestion; the patients will become pale and emaciated, and appear as if they were affected by some fatal visceral disease: at the same time no morbid structure in the region of the stomach or liver can be detected, by the most attentive examination. In some of these cases, the patients have been completely restored to health by a course of the Bath waters.

### *Of Inflammation of the Bowels.*

Of this very formidable disease I have very little to observe. Where the symptoms had been fully formed, the greater number of cases which I have seen have terminated fatally. One case, however, in which the vomiting was of stercoraceous matter, recovered. The chief remedy in this very dangerous disease is bleeding largely, both from the system and topically by leeches. It is very desirable that the inflammation should be subdued, or at least be much lessened, before any active purgative be administered. A purgative during the violence of the inflammation will rarely produce any evacuation, and may even do some injury, by stimulating a part still highly inflamed. Fomentations have been very commonly applied to the belly, and they give some temporary relief. I am inclined to think that cold applications may be useful in assisting to subdue the inflammation; but this I have not hitherto tried. The tobacco glyster, and cold water thrown upon the lower limbs, have in some cases excited the bowels to action, when very powerful purgatives had failed.

*Of Dysentery.*—In this disease, opiate and astringent medicines have sometimes appeared to me to be administered too early. Mild purgative medicines (of which I think castor-oil, upon the whole, the best,) should be administered till the alvine evacuations have become free from mucus and blood, and have recovered in a considerable degree the appearance of a natural fluid motion. Astringent medicines, with opium, may then be directed with much advantage. As there is always an inflammatory condition of the bowels in this disease, leeches may be applied to the seat of the sigmoid flexure of the colon, and the upper extremity of the rectum, with a considerable chance of benefit.

[To be concluded in our next Number.]

